# Supplementary material for: Genomic determinants of antigen expression hierarchy in African trypanosomes
Source: Nature. 2025 Mar 12;642(8066):182–90. doi: 10.1038/s41586-025-08720-w (PMC12137147; doi:10.1038/s41586-025-08720-w)
Supplement: Supplementary file 1 — Uncropped images of Fig. 1b and Extended Data Figs. 4b and 6b,h. [file 41586_2025_8720_MOESM1_ESM.pdf]

nature portfolio

https://doi.org/10.1038/s41586-025-08720-w

Supplementary information

Genomic determinants of antigen expression hierarchy in African trypanosomes

In the format provided by the
authors and unedited

Supplementary Figure 1

	page
Uncropped images of Figure 1b and ED Figure 4b	1
Uncropped images of ED Figure 4b	2
Uncropped images of ED Figure 6b	3
Uncropped images of ED Figure 6h	4

Figure 1b

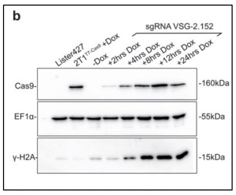

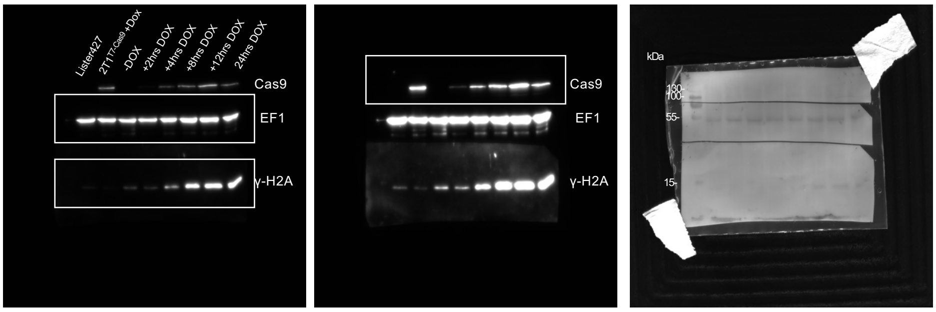

20second exposure (for EF1 and γ-H2A)

2min exposure (for Cas9)

Controls were loaded on the same gel as the test samples.
The membrane was cut in 3 (top for Cas9, middle for EF1 and bottom for γ-H2A detection) prior to primary antibody incubation. The three membranes were exposed together.

Extended data Figure 4b

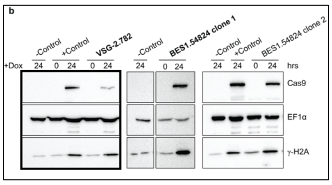

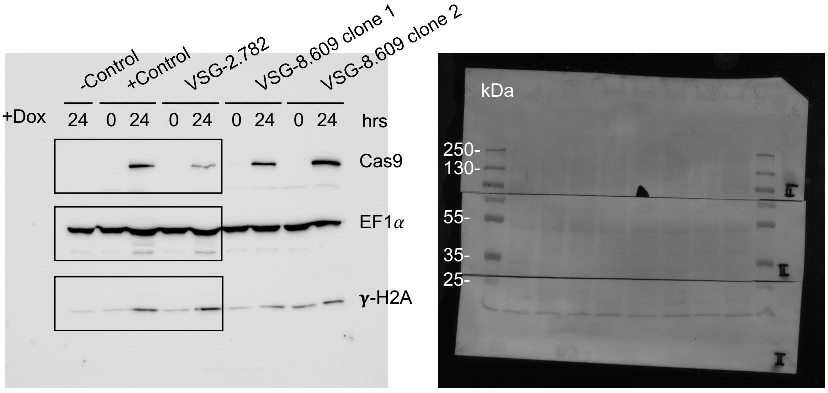

10-second exposure for Cas9, EF1α, **γ**-H2A. All membrane pieces (3) were exposed together.

Extended data Figure 4b

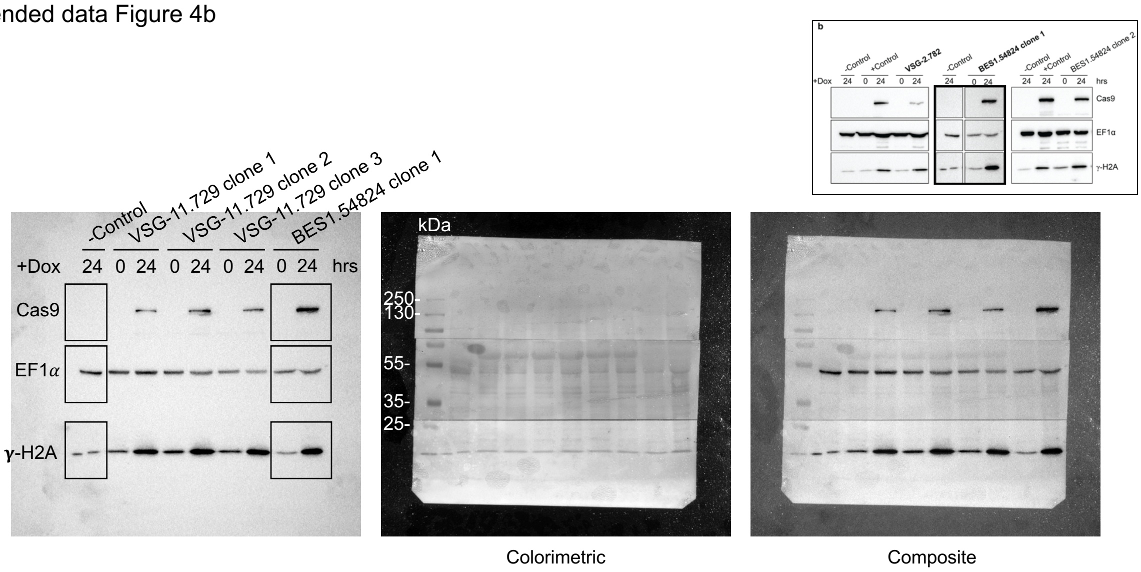

10-second exposure for Cas9, EF1α, γ-H2A. All membrane pieces (3) were exposed together.

Extended data Figure 4b

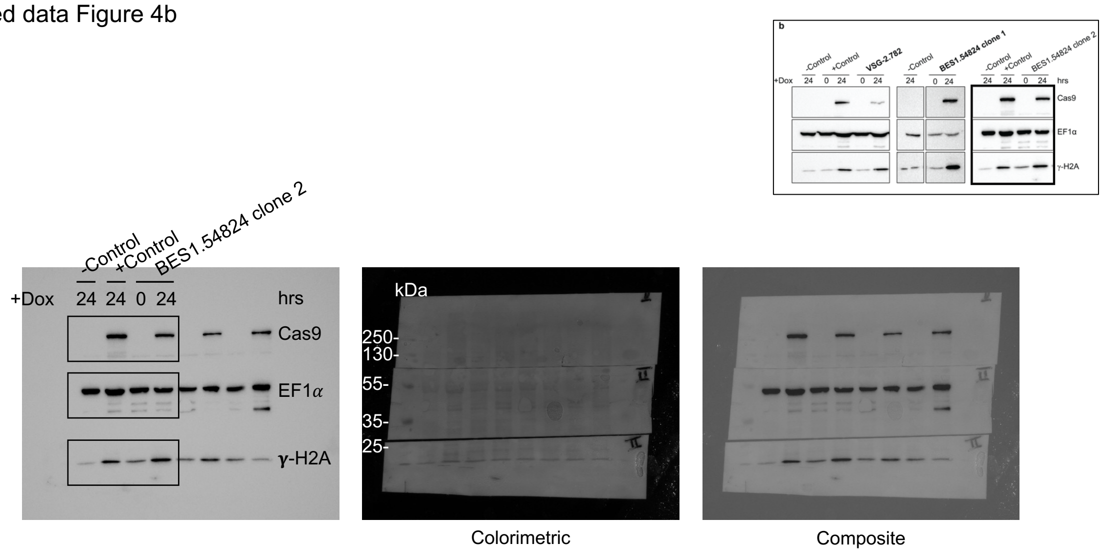

10-second exposure for Cas9, EF1α, γ-H2A. All membrane pieces (3) were exposed together.

Extended data Figure 6b

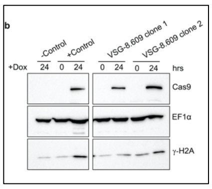

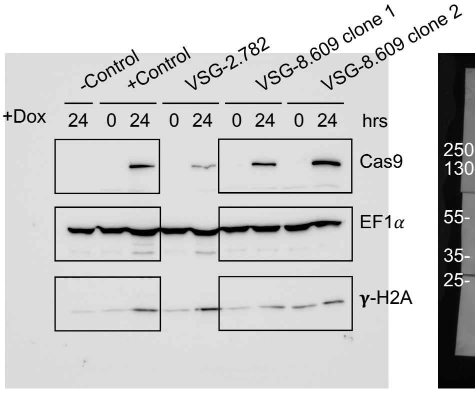

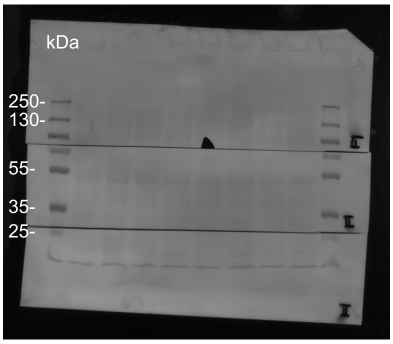

10-second exposure for Cas9, EF1α, **γ**-H2A. All membrane pieces (3) were exposed together.

Extended data Figure 6b

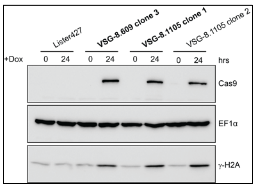

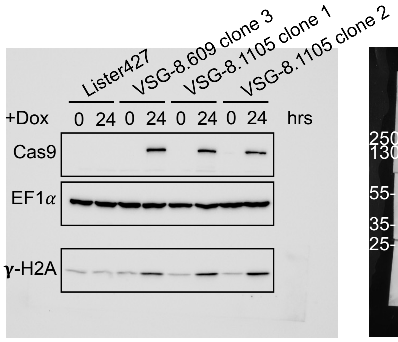

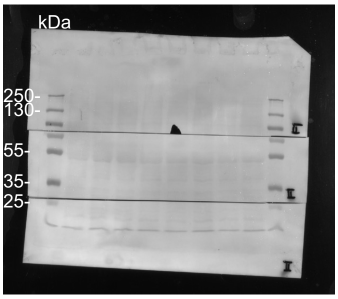

Colorimetric

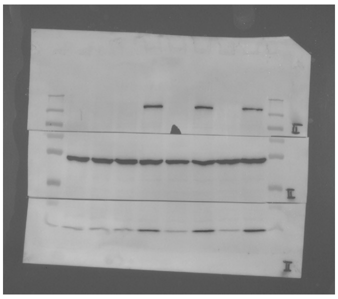

Composite

11.82-second exposure for Cas9, EF1α, **γ**-H2A. All membrane pieces (3) were exposed together.

Extended data Figure 6h

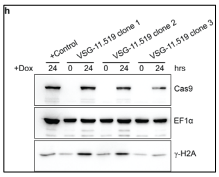

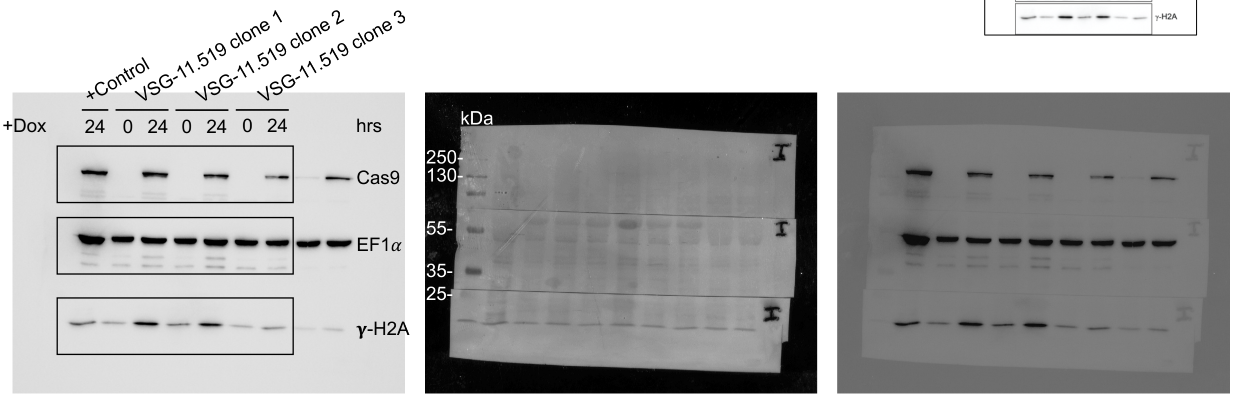

Colorimetric

Composite

10-second exposure for Cas9, EF1α, γ-H2A. All membrane pieces (3) were exposed together.

Extended data Figure 6h

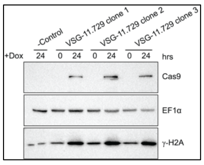

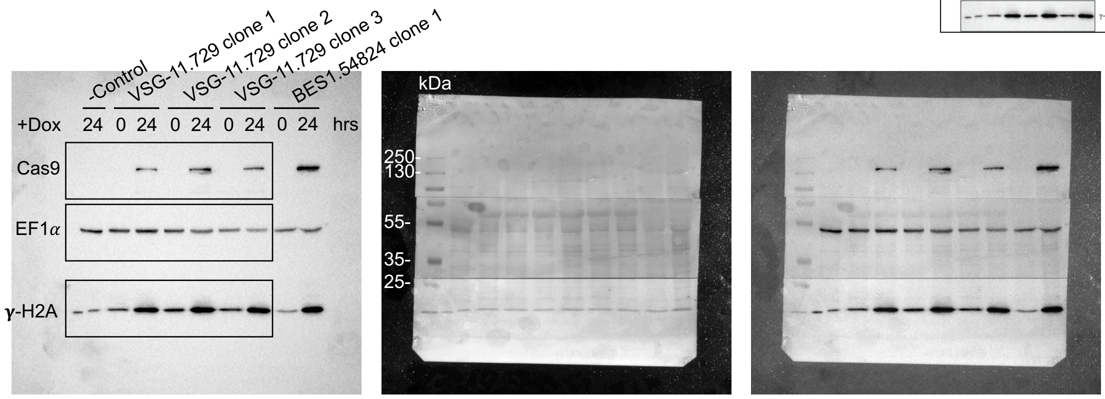

Colorimetric

Composite

10-second exposure for Cas9, EF1α, γ-H2A. All membrane pieces (3) were exposed together.
