# Supplementary material for: Genomic determinants of antigen expression hierarchy in African trypanosomes
Source: Nature. 2025 Mar 12;642(8066):182–90. doi: 10.1038/s41586-025-08720-w (PMC12137147; doi:10.1038/s41586-025-08720-w)
Supplement: Supplementary file 2 — Reporting Summary [file 41586_2025_8720_MOESM2_ESM.pdf]

Corresponding author(s): Siegel
Last updated by author(s): Jan 10, 2025

Reporting Summary

Nature Portfolio wishes to improve the reproducibility of the work that we publish. This form provides structure for consistency and transparency in reporting. For further information on Nature Portfolio policies, see our Editorial Policies and the Editorial Policy Checklist.

Statistics

For all statistical analyses, confirm that the following items are present in the figure legend, table legend, main text, or Methods section.

n/a	Confirmed	
☐	☒	The exact sample size (n) for each experimental group/condition, given as a discrete number and unit of measurement
☐	☒	A statement on whether measurements were taken from distinct samples or whether the same sample was measured repeatedly
☒	☐	The statistical test(s) used AND whether they are one- or two-sided<br>*Only common tests should be described solely by name; describe more complex techniques in the Methods section.*
☒	☐	A description of all covariates tested
☒	☐	A description of any assumptions or corrections, such as tests of normality and adjustment for multiple comparisons
☐	☒	A full description of the statistical parameters including central tendency (e.g. means) or other basic estimates (e.g. regression coefficient) AND variation (e.g. standard deviation) or associated estimates of uncertainty (e.g. confidence intervals)
☒	☐	For null hypothesis testing, the test statistic (e.g. F, t, r) with confidence intervals, effect sizes, degrees of freedom and P value noted<br>*Give P values as exact values whenever suitable.*
☒	☐	For Bayesian analysis, information on the choice of priors and Markov chain Monte Carlo settings
☒	☐	For hierarchical and complex designs, identification of the appropriate level for tests and full reporting of outcomes
☒	☐	Estimates of effect sizes (e.g. Cohen's d, Pearson's r), indicating how they were calculated

Our web collection on statistics for biologists contains articles on many of the points above.

Software and code

Policy information about availability of computer code

Data collection	Software used for data collection (Immunofluorescence analysis):<br>LASX / v3.7.6 software / https://www.leica-microsystems.com/products/microscope-software/p/leica-las-x-ls/
Data analysis	SOFTWARE / VERSION<br>FlowJoTM Software / v10.10.0<br>Graphpad / v9<br>Fiji / v2.0<br>python / v3.10.8<br>IPython / v7.31<br>deepTools / v3.5.4<br>STAR / v2.7.10a<br>BLAST / v2.14.0<br>minimap2 / v2.10<br>seqkit / v2.5.1<br>perl / v.5.32.1<br>perl-bioperl / v1.7.8<br>trinity / v2.15.1<br>seqtk / v1.4<br>cutadapt / v4.3<br>cutadapt / v3.5<br>biopython / v1.81

jupyterlab / v4
matplotlib / v3.6.3
pandas / v1.5.3
numpy / v1.23.5
seaborn / v0.12.2
pygenometracks / v3.8
scanpy / v1.7.2
openpyxl / v3.1.2
scipy / v1.10.1
scSwitchFilter / v1.0.0
bwa / v0.7.17
picard / v3.2.0
umi_tools /v1.1.2
subread /v2.0.1
samtools / v1.17
samtools / v1.20
Protospacer Workbench / v0.1.0 beta
ChemiDoc MP Imaging System / v3.0.1.14
IGV / v2.16.0
deML / v1.1.13
FCS Express software / v7

Hardware:
High performance computing system (HPC) from the Biomedical Center Munich (https://www.compbio.bmc.med.uni-muenchen.de/hpc/index.html)

Operating system:
CentOS 7; with GNU bash, version 4.2.46(1)-release (x86_64-redhat-linux-gnu). Processes were run using SLURM (job scheduling system) version v16.05.2

Workflows and scripts code:
All custom scripts and computational workflows are publicly available at Zenodo (https://doi.org/10.5281/zenodo.10692101). Computational environment files (conda yaml files) to reproduce the software set up used during the analysis are provided. Documentation for reproducing data analysis is provided.

For manuscripts utilizing custom algorithms or software that are central to the research but not yet described in published literature, software must be made available to editors and reviewers. We strongly encourage code deposition in a community repository (e.g. GitHub). See the Nature Portfolio guidelines for submitting code & software for further information.

Data

Policy information about availability of data

All manuscripts must include a data availability statement. This statement should provide the following information, where applicable:

- Accession codes, unique identifiers, or web links for publicly available datasets
- A description of any restrictions on data availability
- For clinical datasets or third party data, please ensure that the statement adheres to our policy

Data availability
The scRNA-seq, RNA-seq, ATAC-seq and BLISS data generated for this project have been deposited in the European Nucleotide Archive and are accessible through ENA study accession number PRJEB72370. The scRNA-seq data published by Müller et al. (doi.org/10.1038/s41586-018-0619-8) used in this project is deposited in the Gene Expression Omnibus and are accessible through GEO Series accession number GSE100896. The Tb427v11 genome assembly is available in Zenodo (https:// doi.org/10.5281/zenodo.10692100).

Research involving human participants, their data, or biological material

Policy information about studies with human participants or human data. See also policy information about sex, gender (identity/presentation), and sexual orientation and race, ethnicity and racism.

Reporting on sex and gender	N/A
Reporting on race, ethnicity, or other socially relevant groupings	N/A
Population characteristics	N/A
Recruitment	N/A
Ethics oversight	N/A

Note that full information on the approval of the study protocol must also be provided in the manuscript.

Field-specific reporting

Please select the one below that is the best fit for your research. If you are not sure, read the appropriate sections before making your selection.

☒ Life sciences ☐ Behavioural & social sciences ☐ Ecological, evolutionary & environmental sciences

For a reference copy of the document with all sections, see nature.com/documents/nr-reporting-summary-flat.pdf

Life sciences study design

All studies must disclose on these points even when the disclosure is negative.

Sample size	Sample size was not statistically predetermined for the individual experiments. The sample size selected offered a good compromise between scale and cost. Due to the low heterogeneity the number of cells analyzed were sufficient for the analysis.
Data exclusions	SL-Smart-seq3xpress data analysis: Cells with less than 500 genes detected, 1000 gene UMI transcript counts were filtered-out.
Replication	All attempts of replication were successful. All scRNA-seq experiments were done in biological duplicates. Cell density measurements were done in triplicates except for growth curves shown in Extended Data Figure 6f, for which only one measurement was taken. No experiments other than those mentioned in the reporting summary were performed.
Randomization	No randomization applied. Since cell sorting was done in different days no randomization was possible.
Blinding	No blinding applied, as we do not perform case / control analyses.

Reporting for specific materials, systems and methods

We require information from authors about some types of materials, experimental systems and methods used in many studies. Here, indicate whether each material, system or method listed is relevant to your study. If you are not sure if a list item applies to your research, read the appropriate section before selecting a response.

Materials & experimental systems

n/a	Involved in the study	
☐	☒	Antibodies
☐	☒	Eukaryotic cell lines
☒	☐	Palaeontology and archaeology
☒	☐	Animals and other organisms
☒	☐	Clinical data
☒	☐	Dual use research of concern
☒	☐	Plants

Methods

n/a	Involved in the study	
☒	☐	ChIP-seq
☐	☒	Flow cytometry
☒	☐	MRI-based neuroimaging

Antibodies

Antibodies used	ANTIBODY / SOURCE / IDENTIFIER<br>Alexa Fluor TM 488-conjugated anti-VSG-2 / The antibody was generated by Pinger et al, 2017 (https://doi.org/10.1038/s41467-017-00959-w), acquired from the Memorial Sloan Kettering, Antibody & Biosource Core Facility (https://www.mskcc.org) and conjugated using the Alexa FluorTM 488 Antibody Labeling Kit (A10235)<br>Mouse anti-CRISPR/ Cas9 7A9-3A3 /Active Motif / Cat # 61978<br>Rabbit anti-gamma-H2A / Dr. Lucy Glover, Institut Pasteur / Glover and Horn, 2012 (doi: 10.1016/j.molbiopara.2012.01.008)<br>Mouse anti-EF1α CBP-KK1 / Merck-Millipore / Cat# 05-235 RRID:AB_309663<br>Goat anti-mouse HRP (used for anti-Cas9 and anti-EF1alpha) / GE Healthcare/ code NA931V<br>Goat anti-rabbit HRP (used for anti-gammaH2A)/ GE Healthcare/ code NA934V
Validation	There was no new antibody generated for this study. All antibody validations have been performed previously, see above.

Eukaryotic cell lines

Policy information about cell lines and Sex and Gender in Research

Cell line source(s)	All Trypanosoma brucei brucei Lister 427 cell lines used and generated in this study are described in the methods section.
Authentication	RNA-seq provided authentication.
Mycoplasma contamination	Mycoplasma contamination check carried our approx. every 3 years - no positive results from those tests to date.

Commonly misidentified lines (See ICLAC register)	No commonly misidentified cell lines were used.

Plants

Seed stocks	N/A
Novel plant genotypes	N/A
Authentication	N/A

Flow Cytometry

Plots

Confirm that:

☒ The axis labels state the marker and fluorochrome used (e.g. CD4-FITC).

☒ The axis scales are clearly visible. Include numbers along axes only for bottom left plot of group (a 'group' is an analysis of identical markers).

☒ All plots are contour plots with outliers or pseudocolor plots.

☒ A numerical value for number of cells or percentage (with statistics) is provided.

Methodology

Sample preparation	For single-cell sorting: 5.0×10^6 cells were harvested by centrifugation at 4°C and washed twice in sterile filtered ice cold 1X TDB. The cells were resuspended in 1ml of ice cold filtered 1X TDB, and stained with 1µg/ml propidium iodide to exclude dead cells.<br>For VSG-2 expression analysis: 1.0×10^6 cells were harvested by centrifugation at 4°C. Cells were incubated in the dark with fluorescently-conjugated anti-VSG-2 diluted 1:500 in HMI-11. Cells were washed three times with 1X TDB and resuspended in 400µl of 1X TDB.
Instrument	For VSG expression analysis FACS Canto (BD Biosciences) was used. For single-cell sorting FACS Fusion II cell sorter (BD Biosciences) was used.
Software	Data collection (VSG expression analysis) and single-cell sorting were performed with the help of FACSDiva software (BD Biosciences). For VSG expression analysis FlowJo TM Software was used.
Cell population abundance	For single-cell sorting the cell population abundance (live cells, singlets) was 11.11%. For VSG-2 expression analysis the cell population abundance (singlets) was between 48.86% and 94.33%, depending on the experiment time point.
Gating strategy	For single-cell sorting:<br>Cell populations were gated to remove cellular debris (FSC-A vs SSC-A), doublets (FSC-A vs FSC-H and SSC-A vs SSC-W), and dead cells (positive for propidium iodide staining).<br>For VSG-2 expression analysis:<br>Cell populations were gated to remove cellular debris (FSC-A vs SSC-A) and doublets (FSC-A vs FSC-H).

☒ Tick this box to confirm that a figure exemplifying the gating strategy is provided in the Supplementary Information.
